# Supplementary material for: Comparative multiomics analysis of cell physiological state after culture in a basket bioreactor
Source: Sci Rep. 2022 Nov 23;12:20161. doi: 10.1038/s41598-022-24687-4 (PMC9686226; doi:10.1038/s41598-022-24687-4)
Supplement: Supplementary file 1 — Supplementary Information 1. [file 41598_2022_24687_MOESM1_ESM.zip › raw data/Transcriptomic raw data/6.Enrichment/1.GO/TE1vsTC1/GSEA/TC1/gsea_report_for_TC1_1656407547197.html]

Report for TC1 1656407547197 [GSEA]


| GS   follow link to MSigDB | GS DETAILS | SIZE | ES | NES | NOM p-val | FDR q-val | FWER p-val | RANK AT MAX | LEADING EDGE || 1 | ANTIGEN\_PROCESSING\_AND\_PRESENTATION(GO:0019882) | Details ... | 21 | -0.62 | -1.36 | 0.000 | 0.189 | 0.174 | 8573 | tags=95%, list=38%, signal=154% |
| 2 | LIPASE\_ACTIVITY(GO:0016298) | Details ... | 22 | -0.19 | -1.15 | 0.000 | 0.742 | 1.000 | 18115 | tags=95%, list=81%, signal=498% |
| 3 | PHOSPHOLIPASE\_ACTIVITY(GO:0004620) | Details ... | 21 | -0.19 | -1.06 | 0.000 | 0.892 | 1.000 | 18115 | tags=95%, list=81%, signal=497% |
| 4 | DNA\_POLYMERASE\_ACTIVITY(GO:0034061) | Details ... | 16 | -0.28 | -1.00 | 0.000 | 1.000 | 1.000 | 16122 | tags=100%, list=72%, signal=356% |
| 5 | CHEMOKINE\_ACTIVITY(GO:0008009) | Details ... | 21 | -0.25 | -1.00 | 0.000 | 1.000 | 1.000 | 13226 | tags=90%, list=59%, signal=221% |
| 6 | G\_PROTEIN\_COUPLED\_RECEPTOR\_BINDING(GO:0001664) | Details ... | 25 | -0.28 | -1.00 | 0.000 | 1.000 | 1.000 | 13226 | tags=92%, list=59%, signal=224% |
| 7 | CHEMOKINE\_RECEPTOR\_BINDING(GO:0042379) | Details ... | 21 | -0.25 | -1.00 | 0.000 | 1.000 | 1.000 | 13226 | tags=90%, list=59%, signal=221% |
| 8 | EXCITATORY\_EXTRACELLULAR\_LIGAND\_GATED\_ION\_CHANNEL\_ACTIVITY(GO:0005231) | Details ... | 20 | -0.35 | -1.00 | 0.000 | 1.000 | 1.000 | 14492 | tags=95%, list=65%, signal=269% |
| 9 | LIPID\_CATABOLIC\_PROCESS(GO:0016042) | Details ... | 16 | -0.25 | -1.00 | 0.000 | 1.000 | 1.000 | 10538 | tags=81%, list=47%, signal=153% |
| 10 | ION\_CHANNEL\_COMPLEX(GO:0034702) | Details ... | 15 | -0.20 | -1.00 | 0.000 | 0.986 | 1.000 | 18020 | tags=100%, list=80%, signal=511% |
| 11 | CATION\_CHANNEL\_COMPLEX(GO:0034703) | Details ... | 15 | -0.20 | -1.00 | 0.000 | 0.897 | 1.000 | 18020 | tags=100%, list=80%, signal=511% |
| 12 | LIPID\_LOCALIZATION(GO:0010876) | Details ... | 21 | -0.21 | -0.97 | 0.521 | 0.850 | 1.000 | 11651 | tags=86%, list=52%, signal=178% |
| 13 | ORGANOPHOSPHATE\_CATABOLIC\_PROCESS(GO:0046434) | Details ... | 35 | -0.15 | -0.90 | 0.521 | 0.948 | 1.000 | 10538 | tags=80%, list=47%, signal=151% |
| 14 | NUCLEOTIDE\_CATABOLIC\_PROCESS(GO:0009166) | Details ... | 27 | -0.15 | -0.84 | 0.521 | 0.972 | 1.000 | 10310 | tags=78%, list=46%, signal=144% |
| 15 | NUCLEOSIDE\_PHOSPHATE\_CATABOLIC\_PROCESS(GO:1901292) | Details ... | 27 | -0.15 | -0.84 | 0.521 | 0.908 | 1.000 | 10310 | tags=78%, list=46%, signal=144% |
Table: Gene sets enriched in phenotype
 **TC1 (2 samples)
 **[plain text format]****
